# Aerosol vaccination with Bacille Calmette-Guerin induces a trained innate immune phenotype in calves

**DOI:** 10.1371/journal.pone.0212751

**Published:** 2019-02-22

**Authors:** Mariana Guerra-Maupome, Dua X. Vang, Jodi L. McGill

**Affiliations:** 1 Department of Veterinary Microbiology and Preventative Medicine, Iowa State University, Ames, Iowa, United States of America; 2 Interdepartmental Microbiology Program, Iowa State University, Ames, Iowa, United States of America; Bose Institute, INDIA

## Abstract

*Mycobacterium bovis* Bacillus Calmette-Guérin (BCG) is a live attenuated vaccine for use against tuberculosis (TB); however, it is known to reduce childhood mortality from infections other than TB. The unspecific protection induced by BCG vaccination has been associated with the induction of memory-like traits of the innate immune system identified as ‘trained’ immunity. In humans and mouse models, *in vitro* and *in vivo* BCG training leads to enhanced production of monocyte-derived proinflammatory cytokines in response to secondary unrelated bacterial and fungal pathogens. While BCG has been studied extensively for its ability to induce innate training in humans and mouse models, BCG’s nonspecific protective effects have not been defined in agricultural species. Here, we show that *in vitro* BCG training induces a functional change in bovine monocytes, characterized by increased transcription of proinflammatory cytokines upon restimulation with the toll-like receptor agonists. Importantly, *in vivo*, aerosol BCG vaccination in young calves also induced a ‘trained’ phenotype in circulating peripheral blood mononuclear cells (PBMCs), that lead to a significantly enhanced TLR-induced proinflammatory cytokine response and changes in cellular metabolism compared to PBMCs from unvaccinated control calves. Similar to the long-term training effects of BCG reported in humans, our results suggest that in young calves, the effects of BCG induced innate training can last for at least 3 months in circulating immune populations. Interestingly, however, aerosol BCG vaccination did not ‘train’ the innate immune response at the mucosal level, as alveolar macrophages from aerosol BCG vaccinated calves did not mount an enhanced inflammatory response to secondary stimulation, compared to cells isolated from control calves. Together, our results suggest that, like mice and humans, the innate immune system of calves can be ‘trained’; and that BCG vaccination could be used as an immunomodulatory strategy to reduce disease burden in juvenile food animals before the adaptive immune system has fully matured.

## Introduction

Evidence from epidemiological studies suggests that previous exposure to *Mycobacterium bovis* Bacillus Calmette-Guérin (BCG), a live attenuated vaccine used to prevent tuberculosis, reduces the risk of childhood mortality due to prevention of sepsis, diarrhea and respiratory infections [[1–4] and reviewed [5, 6]]. It has been suggested that the nonspecific disease resistance induced by BCG is mediated by an enhanced memory-like response of the innate immune system known as ‘trained’ immunity [7, 8].

Trained immunity is induced primarily in myeloid cells (monocytes and macrophages) and natural killer (NK) cells [8–11], after previous exposure to some live vaccines like BCG, measles and yellow fever, as well as to some microbial components of pathogens [11–17], which results in superior cytokine expression and ultimately, enhanced capacity to prevent infection. Mechanistic studies have demonstrated that trained immunity is independent of adaptive immunity [18], and is caused by epigenetic reprogramming and alterations in basal intracellular metabolic pathways, which result in changes in gene expression and cell physiology leading to increased innate immune cells’ capacity to respond to stimulation [9, 10, 16].

Evidence that trained immunity mediates the nonspecific protective effects seen after BCG vaccination came from proof-of-principle experimental studies. In these studies, BCG vaccination of severe combined immunodeficiency (SCID) or recombination-activating gene 1 (rag1)-deficient mice induced protection against subsequent lethal *Candida albicans* (*C*. *albicans*) infection via a mechanism that requires macrophages and proinflammatory cytokine production, both prototypical innate immune components [9, 16, 19]. Mice vaccinated with BCG also show increased resistance to malaria infection, which is associated with increased transcription of antimicrobial proteins compared with nonvaccinated mice [20].

In humans, BCG vaccination leads to an enhanced production of the monocyte-derived proinflammatory cytokines, tumor necrosis alpha (TNFα) and interleukin 6 (IL-6), in response to unrelated bacterial and fungal pathogens (*C*. *albicans*, *Staphylococcus aureus*) [16]; and superior NK cell activity [11]. Moreover, in neonates, intradermal BCG vaccination at birth influences the responses to common vaccine antigens at the systemic level, which appeared to be mediated through activation of T lymphocytes by antigen presenting cells [21]. Similarly, compared with unvaccinated babies, cord blood monocytes from BCG vaccinated infants show increased expression of granulysin and perforin upon stimulation [22]. Taken together, these observations suggest that BCG vaccination has the capacity to train the innate immune system to develop memory-like traits that result in enhanced disease resistance.

The nonspecific protection afforded by parenteral administration of some live vaccines is increasingly appreciated as an aspect of innate immunity that can be exploited for enhancing disease resistance in subjects at high risk from infections [23]. Similar to young human infants, neonatal and juvenile agricultural species are most susceptible to respiratory diseases during the first weeks of life, due to waning maternal immunity and the still-developing neonatal adaptive immune system. In cattle, nearly a quarter of pre-weaned deaths and nearly half of postweaning calf deaths can be attributed to respiratory diseases, with calf pneumonia affecting as many as 20–30% of calves in some operations [24, 25]. Therefore, methods to enhance disease resistance and limit pathogen colonization are highly needed to reduce the risk of respiratory disease in calves, which also serve as valuable animal model for juvenile human respiratory diseases.

The mucosal surface of the respiratory tract represents the principal portal of entry for most human and animal pathogens. It is believed that matching the route of infection with the route of vaccination will provide the best protection against disease [26]. Thus, there are ongoing efforts to evaluate mucosal routes of vaccination (i.e. intranasal or aerosol) for delivery of live respiratory pathogens [27]. BCG vaccination of juvenile calves is a well-described model that has been used previously to explore the adaptive immune response to bovine tuberculosis; and to model the human infant immune response to TB infection [28–31]. Aerosol vaccines have not only the immunological advantage of being able to replicate in the respiratory mucosa and prime the mucosal immune system, but also have practical and logistical advantages [32]. However, few studies have looked at the ability of this route to induce a ‘trained’ immune phenotype at mucosal surfaces, at which there may be a more efficient route of inducing memory-like innate responses against mucosal diseases. Therefore, using the calf model of aerosol BCG vaccination, the objective of this study was to test the hypothesis that both *in vitro* and *in vivo* BCG exposure will ‘train’ bovine innate immune cells to develop memory-like characteristics against unrelated secondary stimuli, and thus can be harnessed as an approach to reduce disease burden in juvenile food animals.

## Materials and methods

### Animal use ethics

All animal procedures were conducted in strict accordance with federal and institutional guidelines and were approved by the Kansas State University Institutional Animal Care and Use Committee (Protocol Number: 27–2956). All the animals in this study were group housed in outdoor pens at the College of Veterinary Medicine, Kansas State University in Manhattan, KS or at the Kansas State University Dairy Farm in Manhattan, KS. Animals had *ab libitum* access to hay, water, and concentrate. No procedures were identified to cause suffering or distress in the animals, and thus no procedures required the use of analgesics or anesthetics. Steps were taken to avoid prolonged restraint during all handling procedures, and antibiotics and analgesics were administered as needed if animals presented with clinical disease independent of the experimental protocol.

For the *in vitro* studies, peripheral blood was collected from a total of six Holstein heifers (one-two years old), that were housed at the Kansas State University Dairy Facility.

For the vaccine study, fourteen, 6-8-week-old Holstein bull calves were randomly assigned to two groups (n = 7 animals/group): vaccinated and unvaccinated group. Clinical signs, including cough, dyspnea, and loss of appetite were monitored daily throughout the study. Body temperature was assessed if animal demonstrated signs of clinical illness. No adverse effects from immunization were observed. Peripheral blood and broncheoalveolar lavage samples were collected at 4 and 12 weeks postvaccination for evaluation of peripheral and mucosal immune responses, respectively. At the end of the study animals were humanely euthanized by barbiturate overdose.

### Bacterial culture

*M*. *bovis* BCG Danish strain was a gift from Dr. Ray Waters at the National Animal Disease Center, USDA. BCG was prepared using standard techniques in Middlebrook 7H9 liquid media (Becton Dickinson, Franklin Lakes, NJ) supplemented with 10% oleic acid-albumin-dextrose complex (OADC) plus 0.05% Tween 80 (Sigma, St. Louis, Missouri). For *in vitro* and *in vivo* experiments, bacteria in log phase were centrifuged (3000 rpm for 15 minutes) and resuspended in cRPMI without antibiotics for *in vitro* studies, or sterile PBS for *in vivo* vaccination studies (see below for additional details).

### Peripheral blood mononuclear cell preparation, cryopreservation and monocyte isolation

Peripheral blood was collected into 2x acid citrate dextrose via the jugular vein. Peripheral blood monocular cells (PBMCs) were isolated from buffy coats by density centrifugation as previously described [33]. Contaminating red blood cells were removed using hypotonic lysis. Cells were washed three times, counted and resuspended in complete RPMI (cRPMI) composed of RPMI-1640 (Gibco, Carlsbad, CA) supplemented with 2 mM L-glutamine, 25 mM HEPES buffer, 1% antibiotic-antimycotic solution, 1% non-essential amino acids 2% essential amino acids, 1% sodium pyruvate, 50 μM 2-mercaptoethanol (all from Sigma, St. Louis, MO), and 10% (v/v) fetal bovine sera (FBS).

PBMCs from the vaccine study where cryopreserved as follows: briefly, PBMCs were resuspended at 2 x10^7^ cells/mL in 1 mL of precooled FBS containing 10% dimethyl sulfoxide (DMSO), and rapidly brought to −80°C in polystyrene containers, which ensured a slow drop in temperature. After 24 h, the cryovials were transferred to a liquid nitrogen tank where they remained until analysis.

Monocytes were isolated using Magnetic Activated Cell Sorting (MACS) according to manufacturer’s instructions. Briefly, PBMCs were resuspended at 10^7^ cells/mL in MACS buffer (0.5% BSA, 2mM EDTA in PBS) and labeled with 10 μg/mL mouse anti-bovine CD14 (Clone CAM36A) from Washington State Monoclonal Antibody center (Pullman, WA) for 20 min at 4°C. Cells were then washed and labeled with anti-mouse IgG1 magnetic beads (Miltenyi Biotech) and purified by positive selection.

### Bronchoalveolar lavage fluid collection, cell isolation and cryopreservation

Broncheoalveolar lavage (BAL) samples were collected from the group of calves in the vaccine study (n = 7/ group) at 4 weeks post immunization. A modified stallion catheter was blindly passed through the nose and advanced through the trachea until lodging in the bronchus. A total of 180 mL of sterile saline was divided into three aliquots. An aliquot was introduced to the lower respiratory tract, followed by immediate suction to obtain lower airway washes. The procedure was repeated twice more. All three aliquots were pooled at the end of the procedure. BAL samples were kept on ice, filtered over sterile gauze, and centrifuged at 200 x g for 10 minutes. Contaminating red blood cells were removed using hypotonic lysis. Cells were washed, counted and cryopreserved as described above.

### *In vitro* training model in bovine monocytes

**Fig 1:**
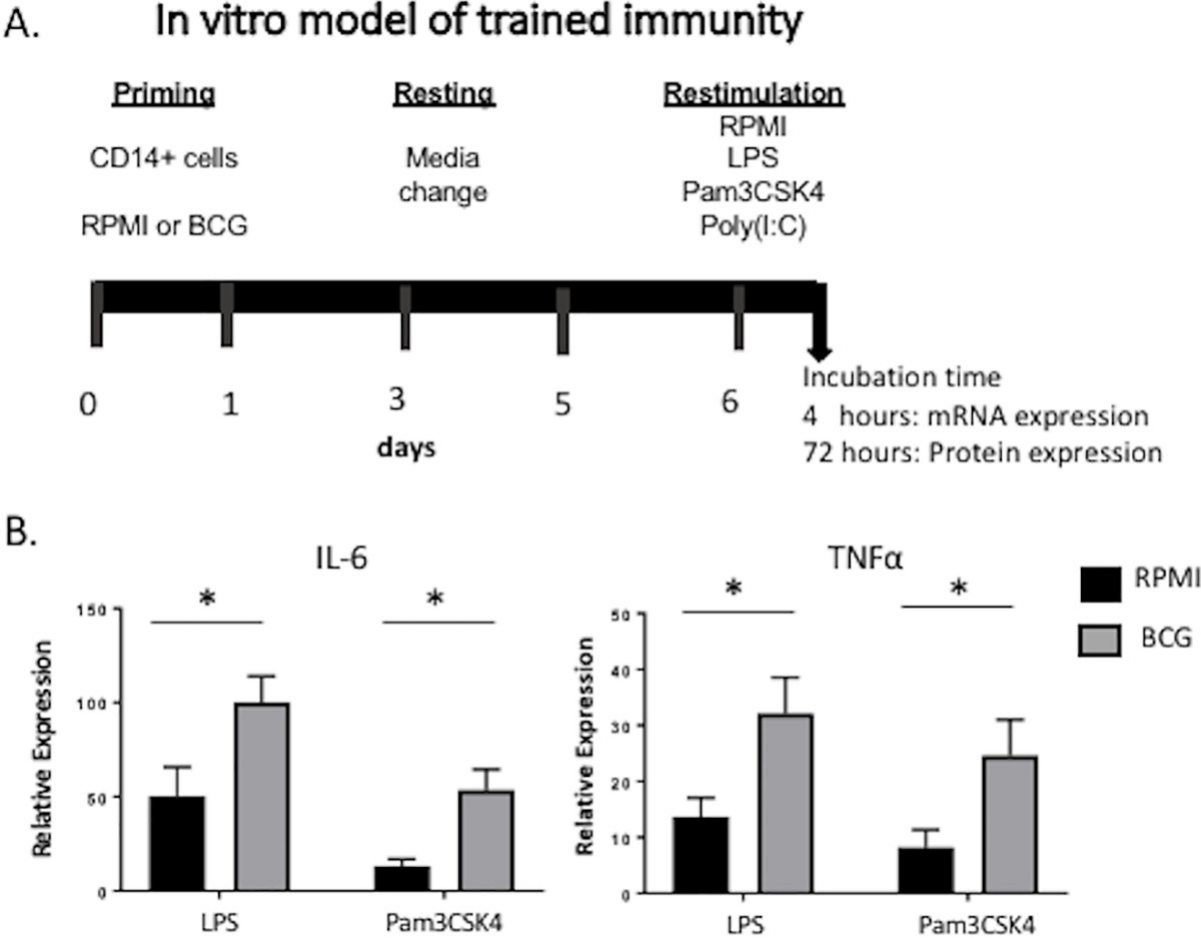
*In vitro* BCG infection of bovine monocytes enhances the expression of proinflammatory cytokines after stimulation with LPS or Pam3CSK4. **(A)** Diagram of the i*n vitro* training model used in bovine isolated monocytes. **(B)** IL-6 and TNFα expression after restimulation for 4 hours. Monocytes isolated from peripheral blood of six healthy heifers were plated at 1x10^6^ cells/mL in a 96-well plate. Cells were infected *in vitro* with BCG at a MOI of 5:1 for 24 hours, washed once with warm cRPMI, incubated for 6 days, and stimulated with LPS, Poly(I:C) or Pam3CSK4 for 4 hours. Uninfected cells (cRPMI) served as negative controls. Changes in gene expression were assessed by RT-PCR. Data represent means ± SEM (n = 6). *p<0.05.

*In vitro* immune training was performed as described previously [16, 34] (**Fig 1A**). Briefly, isolated bovine monocytes were plated in a total volume of 200 μl/well of cRPMI, at a concentration of 1x10^5^ cells/well in flat-bottom, 96-well tissue-culture-treated plates (Falcon). Monocytes were incubated with culture medium only as a negative control. Cells were infected at a 5:1 multiplicity of infection (MOI) with BCG strain Danish for 24 hours at 37° C/5% CO_2_, washed once with warm culture media, resuspended in cRPMI and incubated for 6 days. The media was replaced once, at day 3. After the incubation, the media was removed and cells were re- stimulated with cRPMI, E. coli lipopolysaccharide (LPS; serotype 055:B5; Sigma-Aldrich, 1 μg/mL) or tripalmitoylated lipopeptide (Pam3CSK4; InvivoGen, 10 μg/mL) for 4 hours (mRNA expression) or 72 hours (protein expression). After the indicated times, cells were pelleted, supernatants collected for cytokine measurement and cells lysed with Trizol Reagent (Invitrogen, Life Technologies) for mRNA transcription assessment and stored at -80°C.

### BCG vaccination

For the vaccine study, each calf in the vaccinated group (n = 7) received 1x10^8^ colony-forming units (CFU) of *Mycobacterium bovis* BCG strain Danish via aerosol inoculation as we have previously described [35]. Briefly, the inoculum was nebulized into a mask (Trudell Medical International, London, ON, Canada) covering the nostrils and mouth, allowing regular breathing and delivery of the mycobacteria to the lungs via the nostrils.

### Thawing and *ex vivo* stimulation

For thawing, PBMCs or alveolar macrophages were removed from the liquid nitrogen and thawed in a 37°C water bath for 2 minutes. Once thawed, the cells were rapidly transferred to 15-ml polystyrene tubes containing 8 ml of warm cRPMI to remove DMSO. Finally, the cells were washed twice with complete medium and counted. Cell viability was assessed using Trypan Blue method and found to be >85%. For stimulation experiments, PBMCs or BAL cells (1x10^6^ cells/mL, 1 mL/well) were added to 24-well, tissue-culture-treated plates and rested overnight at 37°C, 5% CO_2_. Cell cultures were then washed once with warm cRPMI, and the cells were stimulated with cRPMI (negative control), LPS, Pam3CSK4 and incubated for 4 hours (mRNA expression) or 72 hours (protein expression). After the indicated times, cells were pelleted, and lysed with Trizol Reagent (Invitrogen, Life Technologies) or cell culture supernatants were collected, and stored at −80°C.

### Real-time PCR

RNA isolation, cDNA preparation and qPCR were performed as described [33]. The primer sequences have been published [36, 37] (**Table 1**). Relative gene expression was determined using the 2^−ΔΔCt^ method, with RPS9 as the reference housekeeping gene [38]. Reactions were performed on an Agilent MX3000P Real-Time PCR machine or Quantstudio 3 Real-Time PCR machine. The following amplification conditions were used: 2 min at 50°, 10 min at 95°, 40 cycles of 15 seconds at 95° and 1 min at 60°, and a dissociation step (15 seconds at 95°, 1 min at 60°, 15 seconds at 95°, 15 seconds at 60°).

**Table 1:** Primers used for RT-PCR.

Gene	Forward (5’-3’)	Reverse (5’-3’)	Accession number^1^
IL-6	CTGAAGCAAAAGATCGCAGATCTA	CTCGTTTGAAGACTGCATCTC	NM_173921.2
IL-1b	TTCTGTGTGACGCACCCGTGC	AGCACACATGGGCTAGCCAGC	X54796
TNFα	CGGGGTAATCGGCCCCCAGA	GGCAGCCTTGGCCCCTGAAG	NM_174088.1
RPS9	CGCCTCGACCAAGAGCTGAGG	CCTCCAGACCTCAGGTTTGTTCC	NM_001101152
TLR2	CGATGACTACCGCTGTGACTC	CCTTCCTGGGCTTCCTCTT	NM_174198.6
TLR4	TGCCTTCACTACAGGGACTTT	TGGGACACCACGACAATA AC	NM_174197.2

^1^ Accession numbers from NCBI database http://www.ncbi.nih.gov.

### ELISAs

The concentration of cytokines in cell culture supernatants was determined after 72 hours of incubation, using bovine commercial ELISA kits for interleukin-1 beta (IL-1β), tumor necrosis factor alpha (TNF-α), and IL-6 (all from Kingfisher Biotech, Inc., St. Paul, MN) according to manufacturer’s instructions.

### Flow cytometry

For flow cytometric analysis, PBMC were added to 96-well round bottom plates (1X10^6^ cells/well). Staining was performed at 4°C. Cells were labeled for 25 minutes with Live/Dead Aqua (Thermo Fisher) and 10 μg/mL of the following primary antibodies: mouse anti-bovine CD14 (clone CAM36A) and CD11b (clone MM10A) from Washington State Monoclonal Antibody center. Cells were washed once, and then incubated for 25 minutes with 0.5 ug/mL of the following secondary antibodies: PeCy7 (IgG1, Biolegend) and APC-Cy7 (IgG2b, Southern biotech). Samples were acquired using a BD LSR Fortessa flow cytometer (BD Biosciences). Data were analyzed using FlowJo (Tree Star Inc., San Carlos, CA).

### Metabolite measurements

The concentration of glucose and lactate in cell culture supernatants was determined after 72 hours of incubation, using Glucose Colorimetric Assay Kit (Biovision Inc.) and Lactate Colorimetric Assay Kit (Biovision Inc.), respectively.

### Statistics

2^-ΔΔCt^ method was used in the statistical analysis of relative gene expression (shown as expression relative to uninfected or unstimulated control samples). Statistical comparisons were performed using One-way ANOVA followed by Tukey’s multiple comparison test. p value below 0.05 was considered statistically significant. All data were analyzed using GraphPad Prism software version v6.0f (La Jolla, CA, USA). Data are shown as means ± standard errors of the means.

## Results

### *In vitro* BCG priming induces trained bovine monocytes, characterized by increased transcription of proinflammatory cytokines

*In vitro* BCG priming induces enhanced proinflammatory cytokine expression in response to unrelated antigens in murine and human monocytes [11, 16, 34, 39]. To test if bovine monocytes have the capacity for innate training, we utilized a traditional *in vitro* protocol of trained immunity (**Fig 1A**) [16, 34]. Circulating bovine monocytes were isolated from peripheral blood and exposed to BCG *in vitro* for 24 hours, rested for 6 days and stimulated with unrelated microbe-associated molecular patterns (LPS, Poly(I:C) or Pam3CSK4). RT-PCR was used to analyze changes in gene expression of IL-6 and TNFα. As shown in **Fig 1B**, bovine monocytes primed *in vitro* with BCG mounted an enhanced response to LPS or Pam3CSK4 compared to non- trained cells, characterized by higher expression of TNFα and IL-6. These results suggest that, similar to observations in humans and mice models [16, 34, 39], *in vitro* BCG priming of bovine monocytes induces increased responsiveness to secondary unrelated stimuli, as measured by expression of the proinflammatory cytokines TNFα and IL-6.

### BCG vaccination of young calves induces enhanced nonspecific production of monocyte-derived proinflammatory cytokines

**Fig 2:**
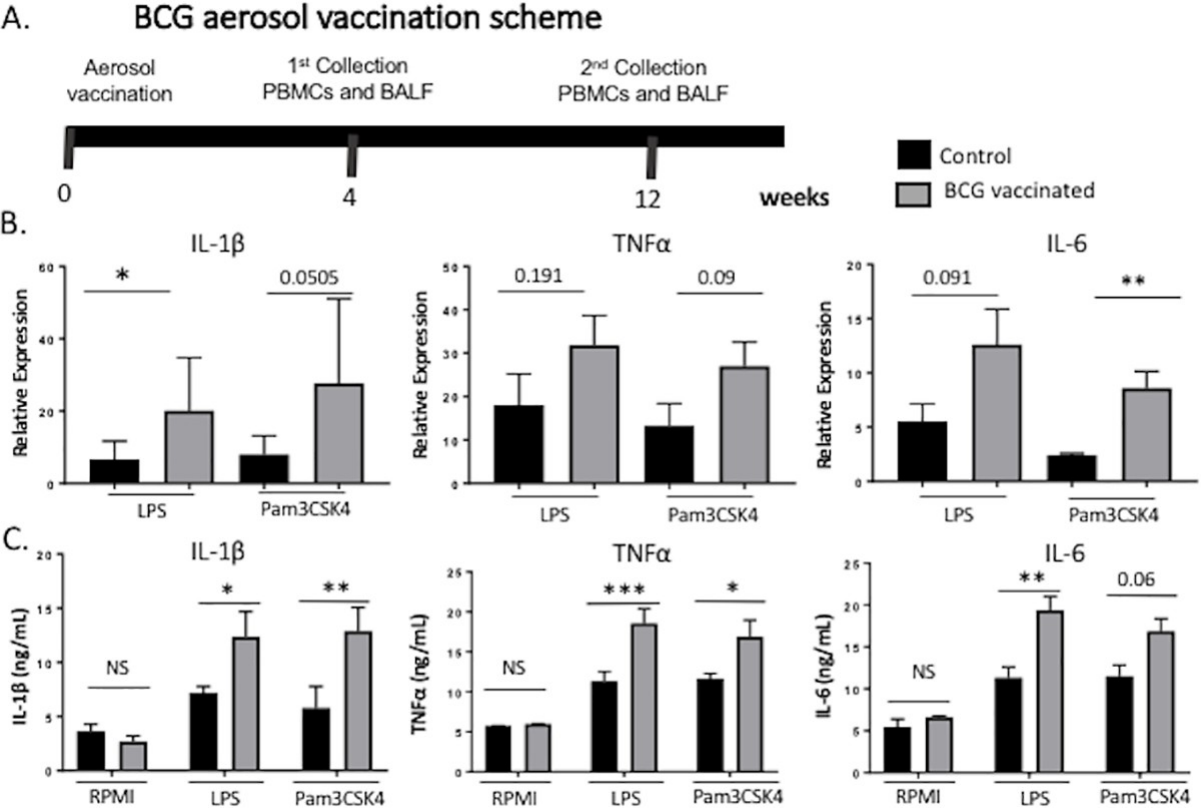
BCG vaccination increased nonspecific production of monocyte-derived proinflammatory cytokines. **(A)** Diagram showing the BCG vaccination schedule in calves. Calves were vaccinated with 1x10^8^ colony forming units (CFU) BCG Danish via aerosol. Peripheral blood was collected four weeks post vaccination from calves in both groups. **(B)** PBMCs isolated from calves after four weeks post vaccination, were stimulated *in vitro* with LPS or Pam3CSK4 for 4 or 72 hours to measure cytokine expression. Proinflammatory cytokine gene expression was assessed by RT-PCR (B), and protein expression was assessed by ELISA on the cell supernatants (C). Data represent means ± SEM. *p<0.05, **p<0.01.

Previous studies have demonstrated that parenteral BCG immunization in healthy adult humans enhances the immune response of peripheral monocytes, macrophages and NK cells upon subsequent *ex vivo* peripheral blood mononuclear cell restimulation [11, 16, 40]. Therefore, we next determined if BCG vaccination also had the capacity to train peripheral mononuclear cells in calves. To explore the possible impact of mucosal immune training on the juvenile immune system, we employed an aerosol route of BCG vaccination for our studies. A total of 14 calves were divided into two groups (n = 7 animals/group). The vaccinated group received 1x10^8^ cfu BCG strain Danish via aerosol inoculation (**Fig 2A**). The remaining calves served as unvaccinated controls. Four weeks after vaccination, PBMCs were isolated and restimulated *in vitro* with LPS or Pam3CSK4 for 4 hours (mRNA expression) or 72 hours (protein expression). As shown in **Fig 2B** and **2C**, PBMC from calves receiving *in vivo* BCG vaccination demonstrated significantly enhanced expression of the monocyte-derived proinflammatory cytokines IL-6, Il-1β and TNFμ, in response to *ex vivo* stimulation with LPS and Pam3CSK4. Together, our results confirm previous reports in human and murine species, showing that BCG vaccination induces an hyperresponsive state in bovine circulating mononuclear cells, characterized by the enhanced production of monocyte-derived proinflammatory cytokines upon secondary stimulation with unrelated antigen. Our results also confirm the ability of BCG to induce memory-like innate immune traits in early life.

### BCG vaccination increases glucose consumption and lactate production by bovine trained monocytes

**Fig 3:**
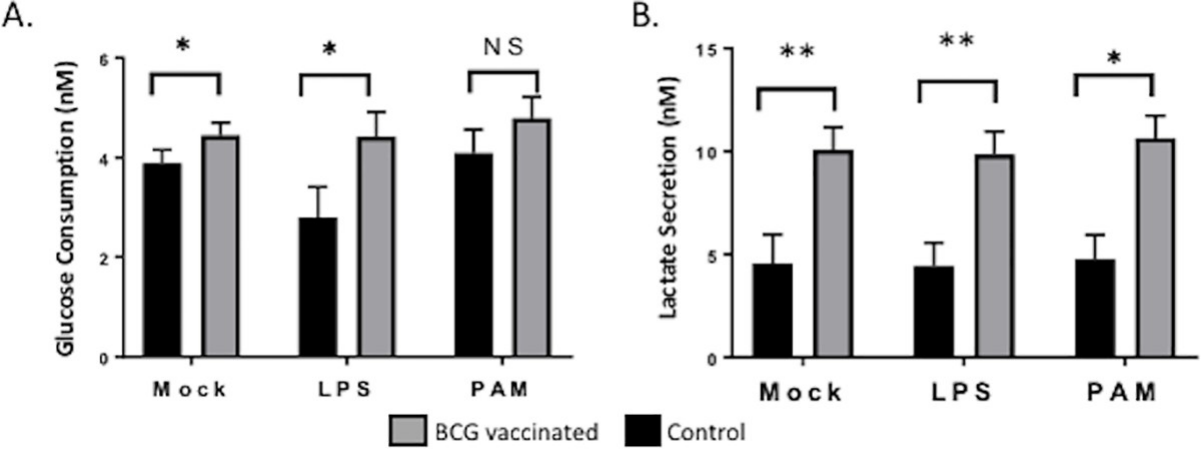
BCG vaccination increases glucose consumption and lactate production in bovine trained monocytes. Peripheral blood was collected four weeks postvaccination from calves in both groups. PBMCs from unvaccinated (grey bars) and BCG vaccinated (black bars) animals were stimulated *in vitro* for 72 hours with LPS or Pam3CSK4, and then glucose consumption (**A**) and lactate concentration (**B)** were measured in the cell culture media. Data represent means ± SEM. Not significant (NS), *p<0.05, **p<0.01, *** p<0.001.

Studies have suggested that one underlying mechanism of trained immunity is linked to specific epigenetic reprogramming that affects immune signaling pathways and cellular metabolism (8–10, 43). We observed that when bovine PBMCs from aerosol BCG-vaccinated animals were stimulated *in vitro* with LPS or Pam3CSK4 for 72 hours, both glucose consumption and lactate production were significantly higher than for stimulated PBMCs from unvaccinated animals (**Fig 3**). Our findings are consistent with reports from human monocytes trained with β-glucan, in which a metabolic switch from oxidative phosphorylation to glycolysis has been described [41]. Thus, results observed here are compatible with the hypothesis that elevated glycolysis is the metabolic basis for the development of trained immunity in BCG-vaccinated calves.

### Aerosol BCG immunization does not alter the number of circulating bovine monocytes or activation marker expression

**Fig 4:**
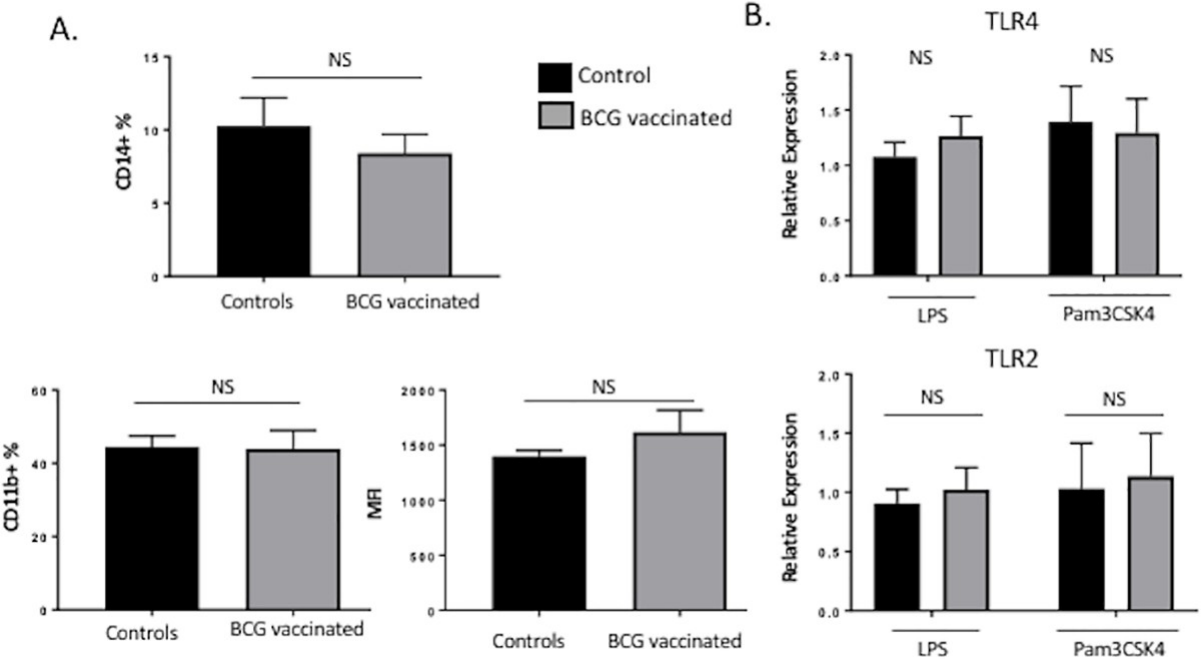
BCG aerosol immunization does not alter the frequency or surface marker expression of bovine circulating monocytes. **(A)** Flow cytometric analysis of circulating CD14+ CD11b+ monocytes isolated 4 weeks after BCG immunization. Average frequencies of CD14+ or CD11b+ cells were analyzed, as were surface expression levels (MFI) of CD11b on cells isolated from both groups of calves (n = 7/group). **(B)** Monocytes were isolated as described in Materials and Methods section and analyzed by RT-PCR for the mRNA expression of TLR2 and TLR4, after 4 weeks of BCG vaccination. Data represent means ± SEM. No significant (NS) differences were observed between treatment groups.

Compared to cells from non-vaccinated individuals, monocytes and NK cells from BCG-vaccinated human adults and infants display slightly increased expression of the surface markers CD14, CD11b and Toll-like receptor 4 (TLR4); but no changes in TLR2 or dectin-1 [11, 16]. To further characterize the phenotype of trained bovine monocytes induced by BCG vaccination, we measured the expression of surface markers by flow cytometry using the gating strategy outlined in S1 Fig. As seen in Fig 4A, no differences were detected in the frequency of CD14+ cells or in the surface expression of CD11b+ between vaccinated and unvaccinated calves. Similarly, TLR4 and TLR2 mRNA expression did not differ between groups at 4 weeks after vaccination (**Fig 4B and 4C**). In accordance with our results, blocking of TLR4 or TLR2 during preincubation with live BCG did not significantly influence the magnitude of trained immunity in mice [42].

### BCG vaccination induces a long-term ‘trained’ phenotype in bovine PBMCs

**Fig 5:**
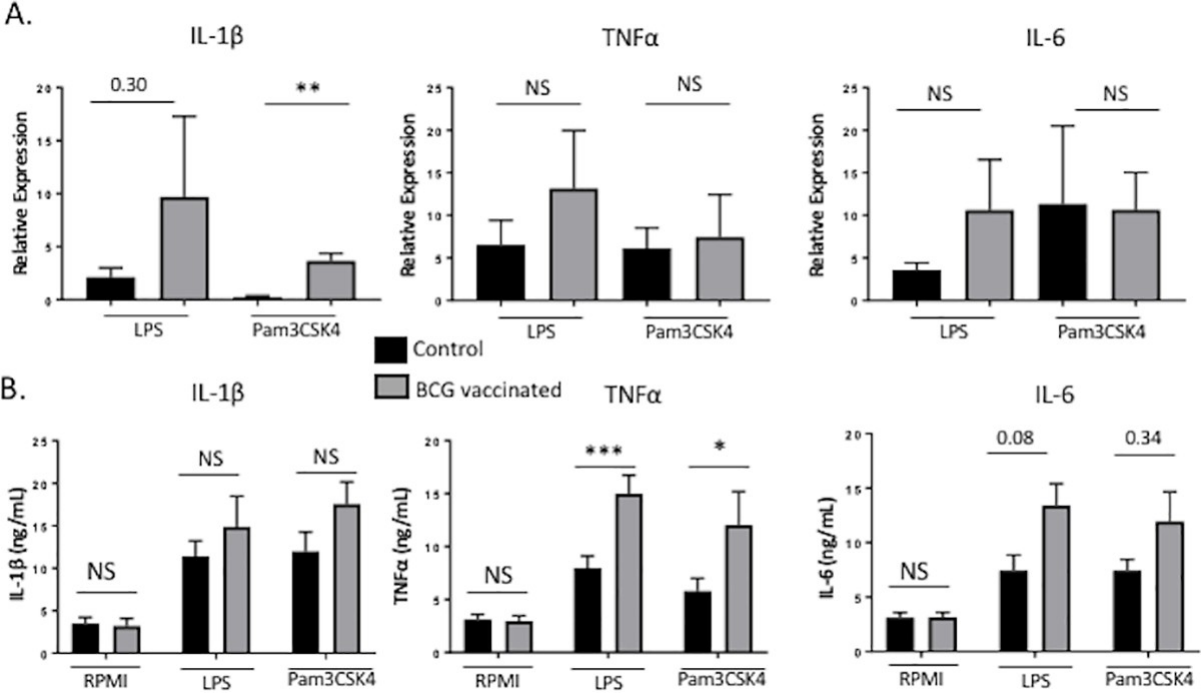
BCG vaccination induces long-term ‘trained’ phenotype in bovine PBMCs. PBMCs were isolated at twelve weeks post vaccination and were stimulated *in vitro* with LPS or Pam3CSK4 for 4 or 72 hours to measure cytokine expression. Proinflammatory cytokine gene expression was assessed by RT-PCR **(A)** and protein expression was assessed by ELISA in the supernatants **(B).** Data represent means ± SEM. No significant (NS), *p<0.05, **p<0.01, *** p<0.001.

The effects of BCG vaccination on innate immune training have been shown to persist for at least 3 months to a year [6, 16, 40]. As seen in **Fig 5**, BCG vaccination also leads to long-term functional changes in bovine peripheral mononuclear cells. PBMC isolated 12 weeks after vaccination displayed enhanced production of proinflammatory cytokines IL-6, IL-1β and TNFα in response to LPS and Pam3CSK4 compared to the unvaccinated group **(Fig 5B).**

### Bovine alveolar macrophages do not adapt a ‘trained’ phenotype in response to aerosol BCG vaccination

**Fig 6:**
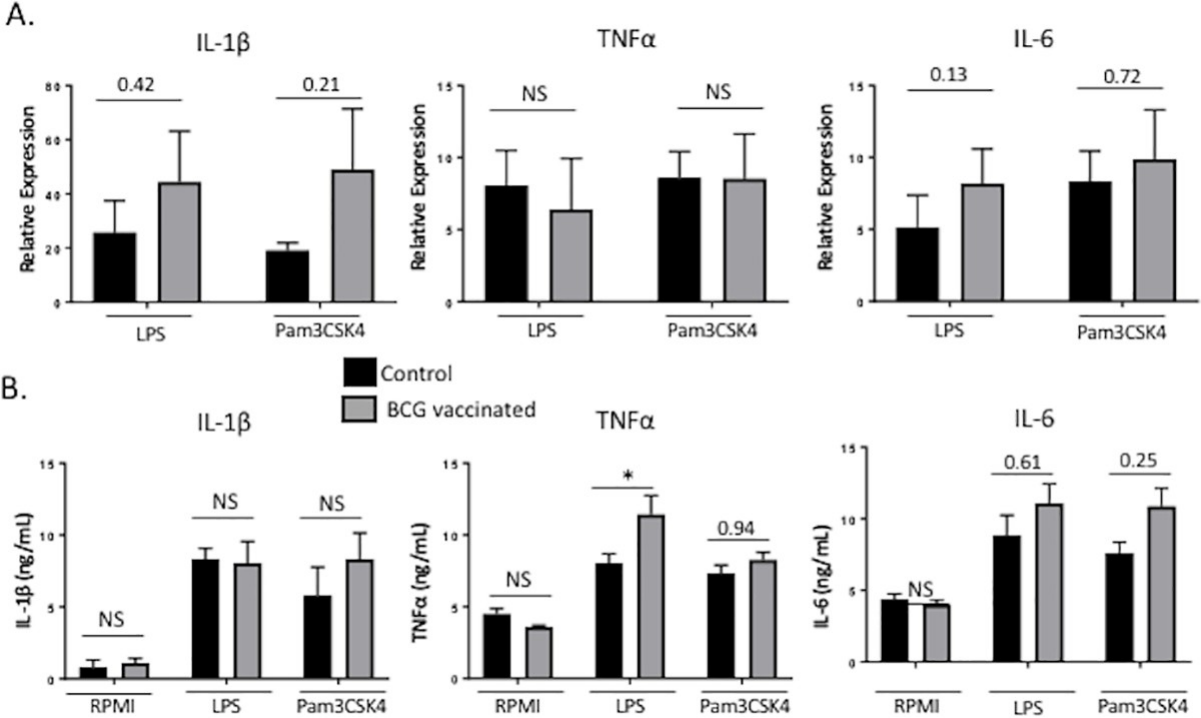
BCG aerosol vaccination does not alter the function of alveolar macrophages. Calves were vaccinated with 1x10^8^ colony forming units (CFU) BCG Danish via aerosol. Peripheral blood was collected four weeks post vaccination from calves in both groups. PBMCs were isolated from calves at four weeks post vaccination and were stimulated *in vitro* with LPS or Pam3CSK4 for 4 or 72 hours to measure cytokine expression. Proinflammatory cytokine gene expression was assessed by RT-PCR **(A),** and protein expression was assessed by ELISA in the supernatants **(B).** Data represent means ± SEM. No significant (NS), *p<0.05.

Respiratory infections are a major cause of juvenile human and animal morbidity and mortality. In the lung, alveolar macrophages (AMs) are the most abundant immune-cell type present under homoeostatic conditions [43]. Considering that BCG influences the functional state of circulating monocytes, and that AMs are critical in immunity to respiratory infections, we hypothesized that aerosol BCG vaccination could induce functional changes at the level of the respiratory mucosa, that could enhance the AMs immune properties. Therefore, we collected BAL samples at 4 weeks post aerosol BCG vaccination. Alveolar macrophages were plated at 1x10^5^ cells/well in 24-well plates and stimulated *in vitro* with LPS or Pam3CSK4 for 4 hours (mRNA expression) or 72 hours (protein expression). As shown in **Fig 6B and 6C**, compared to the control group, aerosol exposure of BCG did not impact gene and protein expression of the proinflammatory cytokines, IL-6, Il-1β or TNFα, in response to *ex vivo* stimulation with LPS and Pam3CSK4. These results indicate that aerosol BCG vaccination does not ‘train’ alveolar macrophages from juvenile calves.

## Discussion

BCG parenteral vaccination has long been known to have beneficial effects against childhood diseases other than TB, and it has recently become clear that these effects arise through a mechanism known as innate training or ‘trained’ immunity [7, 8, 16]. However, the nonspecific effects of BCG have yet to be studied in agricultural species. Here, we demonstrate that, consistent with previous studies in human and mouse models (11, 16, 29, 32, 33), BCG vaccination has the capacity to ‘train’ bovine PBMCs, leading to a significantly enhanced TLR-induced proinflammatory cytokine response compared to PBMCs from control calves (**Fig 2**). Thus, our observations have important clinical relevance, as they suggest that the use of BCG vaccination is likely to confer improved disease resistance, through at least 6 months of age, when the ‘window of susceptibility’ to infection with common diseases in juvenile animals is greatest.

In agreement with data obtained in plants and invertebrates, studies have suggested that trained immunity has a molecular basis as an underlying mechanism, which relies upon specific epigenetic reprogramming that affect immune signaling pathways and cellular metabolism [8–10, 44]. In this study, we identify changes in cellular metabolism induced in bovine PBMCs by aerosol BCG vaccination, demonstrating a switch toward aerobic glycolysis (**Fig 3**). Similarly, earlier studies reported that cell activation and proliferation induced a switch toward aerobic glycolysis in macrophages [45]. Subsequent studies in human monocytes identified a similar metabolic switch after β-glucan priming that involved distinct histone methylations at H3K4 mark with chromatin remodeling at a subset of cytokine promoters that lead to transcriptional programs associated with genes of the mTOR pathway [10, 16, 41]. To our knowledge, our study is the first to observe changes in cellular metabolism induced by BCG training *in vivo*. Our results show that BCG training of bovine monocytes enhances the capacity for cytokine production and identifies an increase in glycolysis as a fundamental metabolic basis for this change.

There is little information on the duration of the transcriptional changes induced by innate immune training. Regarding trained immunity in monocytes, epidemiological studies in children have shown that BCG vaccination induces nonspecific protection that endures at least through early childhood [46]. Considering the short half-life of circulating monocytes, this suggests that epigenetic reprogramming occurs in bone marrow progenitors [17, 47]. Recent findings support this hypothesis, as β-glucan training resulted in favorable adaptations in myelopoiesis. In this study, β-glucan training led to the expansion of hematopoietic stem and progenitor cells (HSPCs), which was associated with IL-1β and GM-CSF (granulocyte-macrophage colony-stimulating factor) signaling, as well as with changes in glucose and lipid metabolism [47]. Identification of core signatures of innate training is thus important, as it allows the better characterization of these functional states and may have consequences in the selection of immunogens, development of adjuvants and vaccine design.

In addition to epigenetic reprogramming, the functional modulation of monocytes, macrophages and NK cells during trained immunity has been linked to an slightly increased expression of PRRs and activation markers crucial for pathogen recognition such as CD14, CD11b, and Toll-like receptor 4 (TLR4), but no changes in TLR2 or dectin-1 [11, 16]. In our study, no differences were detected in the frequencies of circulating monocytes (CD14+), or in the surface expression of CD11b+ between vaccinated and unvaccinated calves. Also, TLR4 and TLR2 mRNA expression did not differ between BCG-vaccinated and control calves 4 weeks after vaccination (**Fig 4A–4C**). In accordance with our results, blocking of TLR4 or TLR2 during preincubation with live BCG did not significantly influence the magnitude of trained immunity in mice [42], suggesting that the ‘trained’ phenotype is likely not simply due to increased expression of PRR or other surface activation markers. A limitation of our study is that we did not investigate the molecular mechanism by which trained immunity was induced in bovine cells. However, epigenetic alterations that facilitate gene transcription has been demonstrated to a mechanism that influences the ‘trained’ immune response to secondary stimulation in other models [9–11, 16, 17]. While this was beyond the aims of the present study, future experiments in cattle should aim to identify the epigenetic program induced after BCG priming, as it is an important question that will allow better characterization of these functional states.

The mucosal surface of the respiratory tract represents one of the principal portals of entry for most human and animal pathogens. Juvenile agricultural species, similar to young human infants, are most susceptible to respiratory diseases during the first weeks of life, as the adaptive immune system develops. Alveolar macrophages (AMs) have an essential role in lung innate immunity and in protecting the host against respiratory diseases [43]. AMs fulfil a variety of functions including, removal of cellular debris, immune surveillance, microbial clearance, responses to infection and the resolution of inflammation. Given that AMs are an important regulator of the local innate response against respiratory pathogens, it is logical to assume that enhanced function may be a more efficient at reducing the risk of respiratory infections and thus, reducing juvenile mortality. However, few studies have looked at the ability to induce an immune ‘trained’ phenotype in AMs. In the current study, we assessed if aerosol BCG vaccination induces functional changes at the level of the respiratory mucosa that could alter the functional properties of AMs. As shown in **Fig 6B and 6C**, our data demonstrate that AM’s from BCG-immunized calves do not adopt a ‘trained’ phenotype in response to *ex vivo* stimulation with LPS and Pam3CSK4. Similar to our results, a low dose of intranasal administration of BCG (TICE strain) in mice has been shown to result in decreased TNF-α mRNA expression and increased IL-10 mRNA expression in AMs [48]. We speculate that the inability to induce a ‘trained’ phenotype in AM’s after BCG vaccination is related to the inherent immunosuppressive properties of AM and the lung environment, as demonstrated by the upregulation of IL-10 in mice [43, 48]. The type of hyperresponsive innate immune state induced by innate training has beneficial effects during host defense, as showed in epidemiological studies, but it could also trigger enhanced tissue damage [49]. It is worth to mentioning, however, that although AMs from mice in the study of Mukherjee *et*. *al*. exhibits an immunosuppressive phenotype, the animals were still protected against a lethal infection with mouse-adapted influenza virus A/Puerto Rico/8/34 (PR8) (H1N1) after intranasal BCG vaccination [48]. Moreover, the nasal route of BCG immunization was more effective than the systemic route against the respiratory challenge. The authors hypothesize that this enhanced resistance is due to increased efferocytosis in animals receiving intranasal BCG vaccination. Thus, limiting immune responses to foreign antigens may be an essential form of adaptation that reduces tissue damage in sites where high pathogen burden occurs, and thus may be an aspect of lung tolerance and resistance to infection. Future studies should be aimed at comparing different routes of immune training (parenteral or mucosal), identifying possible therapies that induce mucosal innate training of AMs, and determining the potential of mucosal ‘trained’ immunity to prevent respiratory infection.

Epidemiological studies show that the nonspecific effects of BCG vaccination are most pronounced in the first year of life, suggesting that trained immunity is most strongly activated during this first year [1, 2]. However, few immunological studies in human infants and neonates have been performed, due to ethical concerns and difficulty obtaining samples. Thus, the full spectrum of innate training and its capacity to promote disease resistance in children has not been fully explored. The calf represents an excellent translational model to investigate the effect of BCG vaccination on susceptibility to common respiratory diseases, as it is a tractable model of the infant immune system and allows for the longitudinal collection of large volumes of peripheral blood and BAL fluid that can be used for cytological, immunological or virological studies. Thus, studies in the calf could benefit both human and animal health and provide information to develop novel vaccination strategies that would more effectively induce both classic adaptive immunity and innate trained immunity to reduce the incidence of common pathogens.

In conclusion, although the effects of BCG on trained immunity have been described before, we provide evidence for the first time that in the young calf, *in vivo* BCG training induces memory-like traits comparable to what has been described in adult human volunteers and mouse models. While BCG vaccination might not be an ideal agent to use for innate training in agricultural species due to regulatory concerns (i.e. skin-testing for monitoring *M*. *bovis* infection), our results warrant further research to identify the microbial components of BCG capable of combining effective induction of adaptive immune responses with elicitation of trained immunity, to enhance diseases resistance against infectious agents in both humans and agricultural species.

## Supporting information

S1 Fig Gating strategy used to identify CD14+ in bovine PBMCs. PBMCs were isolated as described in Materials and methods section. Staining was performed at 4°C. Cells were labeled for 25 minutes with Live/Dead Aqua (Thermo Fisher) and 10 mg/mL of the following primary antibodies: mouse anti-bovine CD14 (clone CAM36A) and CD11b (clone MM10A) from Washington State Monoclonal Antibody center. Cells were washed once, and then incubated for 25 minutes with 0.5 ug/mL of the following secondary antibodies: PeCy7 (IgG1, Biolegend) and APC-Cy7 (IgG2b, Southern biotech). (A) Viable cells with a negative viability dye staining were selected; (B) total live cells were further gated monocyte gate based on FSC-A and SSC-A; (C) CD14+ cells were selected (D) Expression of Mean Fluorescence Intensity (MFI) was assessed. Grey histogram represents fluorescence minus one control (FMO). Data were analyzed using FlowJo (Tree Star Inc., San Carlos, CA).
(DOCX)
