# Supplementary material for: Aerosol vaccination with Bacille Calmette-Guerin induces a trained innate immune phenotype in calves
Source: PLoS One. 2019 Feb 22;14(2):e0212751. doi: 10.1371/journal.pone.0212751 (PMC6386280; doi:10.1371/journal.pone.0212751)
Supplement: S1 Fig — PBMCs were isolated as described in Materials and methods section. Staining was performed at 4°C. Cells were labeled for 25 minutes with Live/Dead Aqua (Thermo Fisher) and 10 mg/mL of the following primary antibodies: mouse anti-bovine CD14 (clone CAM36A) and CD11b (clone MM10A) from Washington State Monoclonal Antibody center. Cells were washed once, and then incubated for 25 minutes with 0.5 ug/mL of the following secondary antibodies: PeCy7 (IgG1, Biolegend) and APC-Cy7 (IgG2b, Southern biotech). (A) Viable cells with a negative viability dye staining were selected; (B) total live cells were further gated monocyte gate based on FSC-A and SSC-A; (C) CD14+ cells were selected (D) Expression of Mean Fluorescence Intensity (MFI) was assessed. Grey histogram represents fluorescence minus one control (FMO). Data were analyzed using FlowJo (Tree Star Inc., San Carlos, CA). (DOCX) [file pone.0212751.s001.docx]

**Aerosol vaccination with Bacille Calmette-Guerin induces a trained innate immune phenotype in calves**

**Mariana Guerra-Maupome^1^, Dua X. Vang^2^, Jodi L. McGill^1^**

**^1^Department of Veterinary Microbiology and Preventative Medicine, Iowa State University, Ames, Iowa, United States of America**

**^2^Interdepartmental Microbiology Program, Iowa State University, Ames, Iowa, United States of America**

**
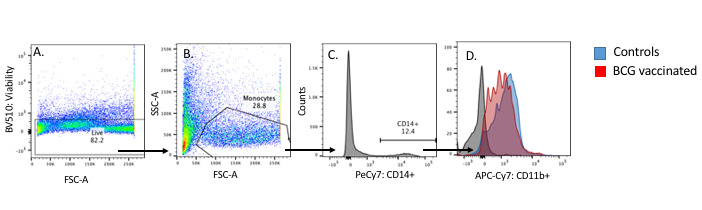
**

**Supplemental Fig. 1. Gating strategy used to identify CD14+ in bovine PBMCs.** PBMCs were isolated as described in Materials and methods section. Staining was performed at 4°C. Cells were labeled for 25 minutes with Live/Dead Aqua (Thermo Fisher) and 10 mg/mL of the following primary antibodies: mouse anti-bovine CD14 (clone CAM36A) and CD11b (clone MM10A) from Washington State Monoclonal Antibody center. Cells were washed once, and then incubated for 25 minutes with 0.5 ug/mL of the following secondary antibodies: PeCy7 (IgG1, Biolegend) and APC-Cy7 (IgG2b, Southern biotech). (A) Viable cells with a negative viability dye staining were selected; (B) total live cells were further gated monocyte gate based on FSC-A and SSC-A; (C) CD14+ cells were selected (D) Expression of Mean Fluorescence Intensity (MFI) was assessed. Grey histogram represents fluorescence minus one control (FMO). Data were analyzed using FlowJo (Tree Star Inc., San Carlos, CA).
